# Factors associated with death anxiety in family caregivers of cancer patients: a systematic review

**DOI:** 10.1186/s12904-026-02127-8

**Published:** 2026-04-30

**Authors:** HuiMin Su, Kuai In Tam, Yang Li

**Affiliations:** 1 Kiang Wu Nursing College of Macau, Complexo de Cuidados de Saúde das Ilhas – Edifício do Instituto de Enfermagem Kiang Wu de Macau, Avenida do Hospital das Ilhas no.447, Coloane, RAEM, Macao Special Administrative Region China; 2 Nursing School of Hunan Normal University, No. 371, Tongzipo Road, Yuelu District, Changsha, Hunan Province China

**Keywords:** Family caregivers, Cancer, Death anxiety, Systematic review

## Abstract

**Objectives:**

This systematic review synthesizes existing literature to identify the current status and key influencing factors related to death anxiety in family caregivers of cancer patients.

**Methods:**

A comprehensive search was conducted across seven databases—PubMed, Embase, PsycINFO, Scopus, Web of Science, MEDLINE, and CNKI on July 26th, 2025, with no time restrictions applied. The quality of all included studies was assessed using the Joanna Briggs Institute critical appraisal tools for cross-sectional studies.

**Results:**

Eighteen studies were included in the systematic review. Based on our analysis, death anxiety among family caregivers of cancer patients may be influenced by the following six categories of factors: (a) personal factors, (b) disease and caregiving-related factors, (c) psychosocial factors, (d) self-regulatory factors, (e) other factors, and (f) actor and partner effects. Several specific protective and risk factors related to death anxiety were also identified.

**Conclusion:**

This review categorizes influencing factors, including protective factors, risk factors, and several contested personal variables, associated with death anxiety. There is a need for longitudinal research to further elucidate the dynamic nature of death anxiety over time. Future studies should adopt a dyadic perspective encompassing both cancer patients and their family caregivers to provide comprehensive insights for healthcare professionals and facilitate the development of effective interventions targeting death anxiety.

**Supplementary Information:**

The online version contains supplementary material available at 10.1186/s12904-026-02127-8.

## Background

Cancer remains a major societal, public health, and economic challenge in the 21 st century, accounting for nearly one in six deaths globally (16.8%) and one in four deaths (22.8%) from non-communicable diseases (NCDs) [1]. According to the latest estimates from the International Agency for Research on Cancer (IARC), there were approximately 20 million new cancer cases and 9.7 million cancer-related deaths worldwide in 2022 [2]. Family caregivers of cancer patients play a critical role in addressing the direct needs of patients, and witnessing the full spectrum of the disease trajectory, from diagnosis and treatment to end-of-life [3]. Family caregivers refer to individuals who provide care for patients ranging from daily living activities to psychological and social aspects, serving as the primary source of unpaid assistance for cancer patients [4]. The prolonged and distressing experience of caring for a loved one with a life-threatening illness exposes caregivers to persistent death-related challenges that increase psychological morbidity and heighten the risk of death anxiety [5–8].

In 2007, the North American Nursing Diagnosis Association (NANDA) formally recognized death anxiety as a nursing diagnosis [9]. Death anxiety (DA) refers to a state of discomfort, worry, and fear arising from an individual’s awareness of death or encounters with dying [9]. Evidence suggests that death anxiety is prevalent among family caregivers [10–12], in some cases surpassing the levels reported by cancer patients themselves due to caregivers’ frequent exposure to death-related cues [13]. Specifically, relevant studies have shown that 73% of caregivers in different regions have a high level of death anxiety [8, 14]. Furthermore, some studies have also indicated that higher levels of death anxiety are associated with an increased risk of psychological disorders such as depression, generalized anxiety, reduced well-being, and diminished quality of life [10]. This negative psychological state can negatively affect caregiving [5, 6], including reduced communication about end-of-life preferences, hindered palliative care engagement, and compromised quality of life and dying experience for patients [15, 16].

Given the detrimental effects of death anxiety, increasing scholarly attention has been directed toward understanding and addressing death anxiety among caregivers in recent years.

Current evidence suggests that factors such as socio-demographic characteristics, disease-related variables, and psychosocial factors contribute to variations in death anxiety among caregivers [10, 17]. Higher levels have been associated with female gender [18], lower educational attainment [19], and greater symptom burden in patients [17], while protective factors include higher caregiver self-efficacy [8] and greater social support networks [20]. Lower caregiver quality of life is also a significant risk factor [21].

Therefore, death anxiety among family caregivers of cancer patients deserves increased clinical and research attention, as it affects not only caregivers’ psychological well-being but also the quality of patient care. Despite growing scholarly interest, there is still no comprehensive review synthesizing the factors associated with death anxiety in this population. This systematic review aims to fill this gap by critically evaluating and summarizing evidence from quantitative studies on the sociodemographic, psychological, and social correlates or predictors of death anxiety among family caregivers of cancer patients.

## Methods

This systematic review was conducted according to the Joanna Briggs Institute (JBI) methodology for systematic reviews of etiology and risk and was reported according to the Preferred Reporting Items for Systematic Reviews and Meta-Analyses [22, 23]. The study protocol was registered in PROSPERO (Registration ID: CRD420251076224).

### Inclusion criteria

#### Participants (population)

Adult family caregivers of cancer patients.

#### Exposure of interest

Death anxiety.

#### Outcomes

Factors related to death anxiety.

#### Types of studies

Observational studies (cross-sectional or longitudinal studies) reporting on factors or associations affecting death anxiety among family caregivers of cancer patients.

### Exclusion criteria

1. Did not focus on family caregivers of cancer patients or did not assess death anxiety as a primary outcome; (2) Were non-original (e.g., conference abstracts, case studies, commentaries, or editorials; (3) Lacked full-text availability; or (4) Were not published in English or Chinese.

### Search strategy

A comprehensive literature search was conducted across seven databases: PubMed, Embase, PsycINFO, Scopus, Web of Science, MEDLINE, and China National Knowledge Infrastructure (CNKI). The search was completed on July 26th, 2025, with no time restrictions on the year of publication.

The search strategy combined both Medical Subject Headings (MeSH) and free-text terms. Keywords include concepts related to death anxiety and death-related distress (e.g., “death anxiety”, “death depression”), caregivers (e.g., “caregivers”, “family caregivers”, “carers”), and cancer (e.g., “cancer”, “neoplasms”, “malignancy”). The complete search queries for each database are provided in Supplementary File 1.

### Study selection

Two reviewers (HS, LY) independently conducted the search and initial screening. Duplicate references were removed using EndNote. Any discrepancies were resolved by discussion; if consensus was not achieved, a third reviewer (KT) adjudicated.

### Quality appraisal

Quality appraisal was performed using the JBI Critical Appraisal Checklist for Analytical Cross-Sectional Studies [22]. This tool consists of eight questions with response options of Yes, No, Unclear, or Not applicable [22]. All appraisal scores were converted to percentages. Studies with “Yes” responses for ≥ 70% of the questions were considered to have a low risk of bias; scores of 50%–69% indicated a moderate risk of bias; and scores ≤ 49% indicated a high risk of bias [24]. Two reviewers (HS & YL) conducted the appraisal independently, with discrepancies resolved through discussion and, if necessary, arbitration by a third reviewer. The results of the appraisal informed the interpretation of findings but were not used as exclusion criteria.

### Data extraction

Two reviewers independently extracted data using a standardized form. Extracted information included: author(s) and year of publication, country of study, family caregivers sample size, study design, cancer type/stage, instrument(s) used to measure death anxiety, correlates or predictor variables analyzed, mean/total death anxiety score, death anxiety outcomes in family caregivers. In case of questions or discrepancies during data extraction, a third reviewer was consulted to resolve the issues after reviewing the original article.

### Data synthesis

Although the included studies reported quantitative data, a meta-analysis was not appropriate due to considerable heterogeneity in study designs and outcome measures. Different instruments (e.g., Templer’s Death Anxiety Scale, Thorson–Powell’s Death Anxiety Scale, and the Collett–Lester Fear of Death Scale) were used, and results were variably reported (e.g., scores out of 15, 100, or 140; as medians or means), without a consistent definition of “high-level death anxiety”, which further limited the comparability of outcomes. These methodological discrepancies precluded meaningful statistical pooling. Therefore, a narrative synthesis was conducted, in line with PRISMA 2020 recommendations. [23].

## Results

The initial comprehensive search yielded 1,317 articles. After the removal of 563 duplicates, 754 records remained for title and abstract screening. Of these, 650 studies were excluded as irrelevant, leaving 104 articles for full-text review. No full-text articles were unavailable. Consequently, 104 articles underwent full-text assessment. Of these, 86 were excluded for the following reasons: conference articles (*n* = 2), case studies or reviews (*n* = 63), book chapters (*n* = 1), publications not in English or Chinese (*n* = 2), and not relevant to topic (*n* = 18).

**Fig. 1:**
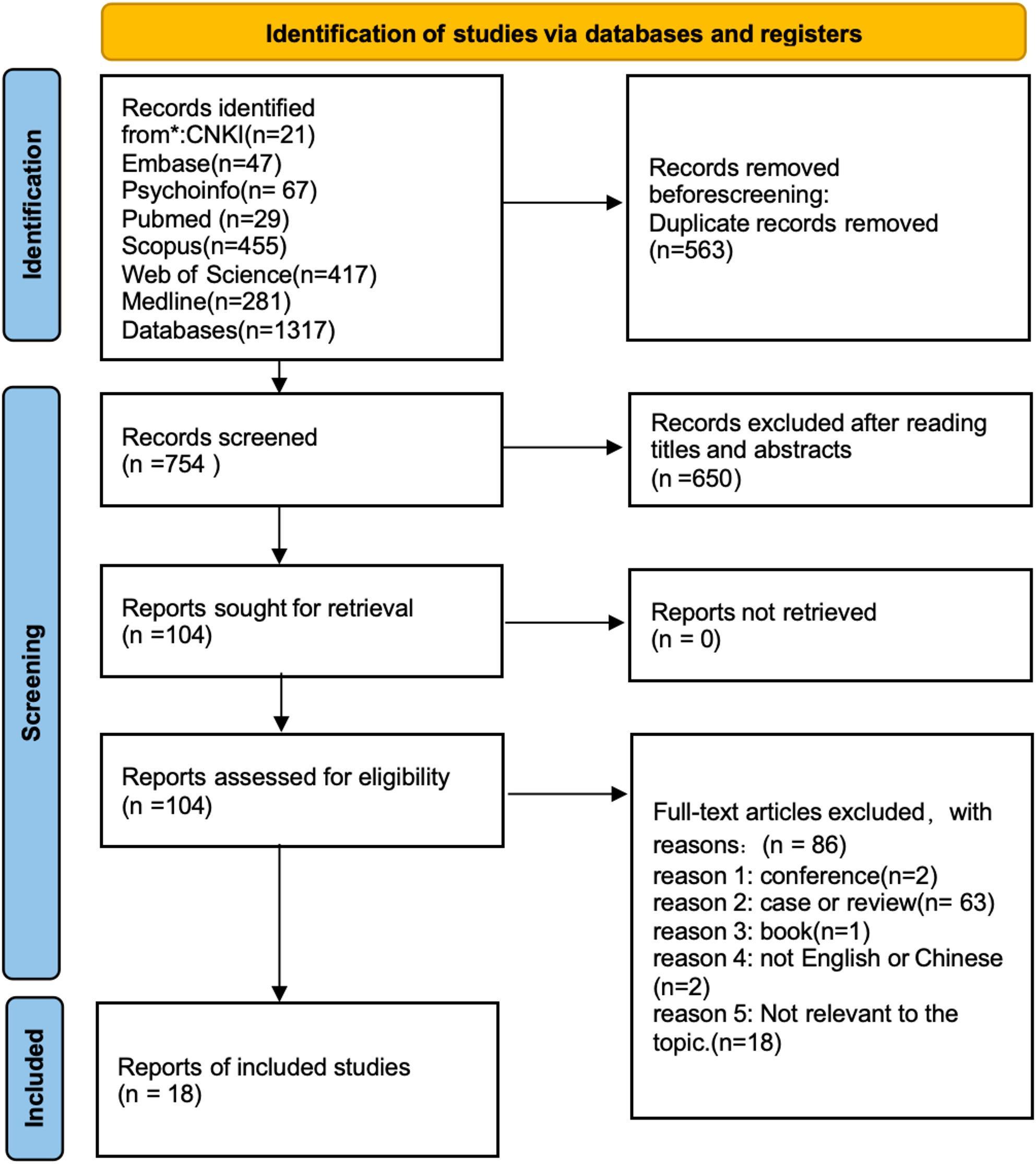
Flow Diagram

Ultimately, 18 studies met the eligibility criteria and were included and analyzed in the systematic review (Fig. 1). All included studies (*n* = 18) satisfied the majority of the JBI critical appraisal criteria and were considered to be of acceptable methodological quality (Supplementary File 2).

This systematic review incorporated a total of 18 studies. Table 1 presents the characteristics of the included studies, including author, publication year, country, sample size of family caregivers, study design, cancer type, disease stage, death anxiety scale used, total death anxiety score, effects on death anxiety/correlations between death anxiety and other variables, and death anxiety outcomes in family caregivers.

**Table 1:** Study characteristics

Author/Year/Country	Sample size of family caregivers/Study design	Cancer Type	Disease staging	DA scale/Total score of DA	Effect on DA/DA Correlate with others variables	DA outcomes in family caregivers
Qian (2022)/China [8]	N=228/cross-sectional	Mixed	Terminal stage	TDAS/8.23±2.56	FC demographic:Female↑, Have religious beliefs↓,High level of education↓,Low income↑,History of physical trauma↑,Have chronic disease↑,Neurotic personality↑,Perceived Social Support↓,Self efficacy↓,Patients' demographic:Lower medical insurance reimbursement ↑,Have recurrence experience↑,Expected survival period ≤4 week↑, Receive palliative care↓,Patient's condition is stable↓,Rescue experience↑;	↑: Neurotic personality (r=0.466, P < 0.01),Female (P=0.007),Low income (P=0.002),Lower medical insurance reimbursement (P=0.017).History of physical trauma (P=0.004),Presence of chronic disease (P<0.001),Recurrence experience (P<0.001),Expected survival period≤4 weeks (P<0.001),Prior rescue experience (P<0.001) ↓: Extraversion personality (r=−0.328, P<0.01),Perceived social support (r=−0.505, P<0.01),Self-efficacy (r=−0.657, P<0.01),Having religious beliefs (P<0.001),Higher education level (P=0.003),Receiving palliative care (P<0.001),Stable patient condition (P=0.005).
Liu et al., (2021)/China [12]	N=205/cross-sectional	Gastric cancer	NA	TDAS/37.94±6.92	FC demographic: Female↑,≤40 years old↑, Unhealthy↑,Have bereavement experience↑,Have chronic disease↑, Self-regulatory fatigue↑,Positive aspects↓.Patients’ demographic:Reoccurrence or metastasis at a distant location↑,ADL ≤40↓.	↑: Female (P<0.001),Age ≤40 years (P<0.001),Poor health status(P<0.001),Experience of bereavement (P<0.001),Presence of a chronic disease (P<0.001),Distant recurrence or metastasis of cancer (P<0.001).↓: Positive aspects (r=−0.580, P<0.01),ADL (Activities of Daily Living) score ≤40 (P<0.001).
Alkan et al. (2020)/Turkey [14]	N=426/cross-sectional	Mixed	I-IV	TDAS/NA	FC demographic:Female↑,Low income↑, Unemployment↑,Have sibling↑,Posttraumatic growth↑.Patient demographic:Older patients↑	↑: Posttraumatic growth (r=0.15, P=0.001).Female gender (P<0.001),Low income (P=0.02),Unemployment (P<0.001),Having a sibling (P=0.01),Caring for older patients (P=0.03).
Soleimani et al. (2017)/Iran [15]	N=330/cross-sectional	Mixed	I-IV	TDAS/46.7±10.7	FC Demographic:Female↑, Age↓, Unemployment↑, High school education or above↑, Income above poverty range↑,As the daughter of the patient↑,All daily prayers in Mosque↓,having family as a main source of income↑,QoL↓.Patient demographic:Radiation therapy↑.	↑: Female (P<0.001),Unemployment (P=0.004),Educational attainment of high school or above (P<0.05),Income above the poverty line (P=0.001),Being the patient’s daughter (P=0.001),Having family as the main source of income (P=0.001), Receiving radiation therapy (P<0.05).↓: QoL (r=−0.41, P<0.01),Age (r=−0.19, P<0.05),Performing all daily prayers in a mosque (P<0.001).
Ying et al. (2024)/China [17]	N=588/cross-sectional	Mixed	III-IV	TDAS/7.92±2.68	QoL↓, Trait anxious personality↑, Social support↓,FC demographic: Female↑,Longer care duration↑,Have religious beliefs↓.Patient factors：Complete self-care↓,Not receiving treatment↑,Have cancer pain↑.	↑: Anxious personality trait (r=0.622, P<0.01),Female gender (P=0.008),Longer caregiving duration (P=0.006),Not receiving active treatment (P=0.008),Presence of cancer-related pain (P=0.006).↓: QoL (r=−0.666, P<0.01),Perceived social support (r=−0.672, P<0.01),Having religious beliefs (P=0.009),Patient ability to perform complete self-care (P=0.007).
Walbaum et al. (2024)/Germany [18]	N=140/cross-sectional	Mixed	IV	DADDS-CG/39.2 ±18.5	FC demographic:Female↑,High school diploma or higher↓	↑: Female (P=0.002).↓: Educational attainment of a high school diploma or higher (P=0.02).
Xie et al. (2025)/China [19]	N=220/cross-sectional	Mixed	I-IV	CL-FODS/104.27±21.02	FC demographic:60–78 years old↑,Primary school or below↑,As the patient's spouse↑, Married↓, Death education↓, County/Town↑, Fully capable of paying for medical expenses↓, Positive coping style↓, Social support↓.Patients’ demographic:Liver cancer↑, IV Tumour stage↑,Able to take care of oneself↓,Patients' fear of death↑.	↑: Age 60–78 years (P=0.021),Education level of primary school or below (P<0.001),Being the patient’s spouse (P<0.001),Residing in a county or town (P<0.001),Liver cancer diagnosis (P=0.004),Tumor stage IV (P=0.011),Patients’ fear of death (P<0.001).↓: Positive coping style (r=−0.42, P<0.001),Social support (r=−0.27, P<0.001).Being married (P<0.001),Receipt of death education (P=0.033),Full ability to pay for medical expenses (P=0.005),Patient being able to perform self-care (P=0.001).
Uslu-Sahan et al. (2019)/Turkey [20]	N=200/cross-sectional	Gynecologic Cancer	IV	TPDAS/46.83±16.88	Perceived social support↓	↓: Perceived social support (r=0.341, P＜0.001).
Sherman et al. (2010)/USA [21]	N=36/cross-sectional	Mixed	Advanced	DAQ/Median 22±6	QoL↓	↓: QoL (r=−0.43, P＜.001).
Bu et al. (2024)/China [25]	N=292/cross-sectional	Mixed	III-IV	TDAS/11.84±3.59	QoL↓,FC Demographic:Death education↑,Lack of support from others↑,Longer duration of illness↑.Patient factors：Patients’DA levels↑	↑: Perceived lack of support from others (P<0.001),Longer duration of illness (P<0.001),Higher patient DA levels (P=0.004). Receipt of death education (P=0.023).↓: QoL (β=−0.565, P<0.001).
Li et al. (2024)/China [26]	N=588/cross-sectional	Mixed	III-IV	TDAS/7.92±2.68	QoL↓,Social support↓,Trait anxious personality↑.	↑: Trait anxious personality (r=0.622, P＜0.01).↓: QoL (r=−0.666, P＜0.01),Social support (r= −0.672, P＜0.01).↗: Trait anxious personality partially mediated the relationship between QoL and DA (indirect effect β=−0.08, P< 0.01). Social support moderated both the antecedent and subsequent segments of the mediating paths of “QoL→trait anxious personality→DA” and the direct relationship between QoL and DA.Social support↓, the mediating effect coefficient of trait anxious personality was higher at 0.25 (95% CI: 0.059–0.182.059.182).Social support↑, the mediating effect coefficient of trait anxious personality was 0.11 (95% CI: 0.029–0.072).
Soleimani et al. (2016)/Iran [27]	N=326/cross-sectional	Mixed	NA	TDAS/Women: 49.52±9.02Men: 42.82±8.55	Female↑	↑: Female (P<0.05).
Lau et al. (2018)/China [28]	N=173/cross-sectional	Lung cancer	I-IV	TDAS/7.65±3.58	Fc’ Dependency↑, Achievement↑, Self‐control↓, QoL↓.Patients’ achievement↑, Dependency↑, Self control↓.Patients' QoL↓.	↑: Caregiver achievement (r=0.21, P<0.01),Caregiver dependency (r=0.38, P<0.001),Patient achievement (r=0.16, P<0.05),Patient dependency (r=0.38, P<0.001)↓: Caregiver self‑control (r=−0.24, P<0.01),Caregiver QoL (r=−0.45, P<0.001),Patient self‑control (r=−0.28, P<0.001),Patient QoL (r=−0.28, P<0.001).
Braun et al. (2021)/USA [29]	N=52/cross-sectional	Brain tumor	I-IV	TDAS/6.08±2.79	Patients’ Fear of cancer recurrence↑	↓: Patients’ FCR(β=0.603, P＜0.05).
Willis et al. (2023)/USA [30]	N=67/cross-sectional	Brain tumor	I-IV	DADDS-CG/34.81±18.96	Caregivers demographic:Female↑.Patients’ demographic:Higher cancer stage↑.	↑: Female (P=0.03),Higher cancer stage (P=0.04).
Eraslan and ILhan (2023)/Turkey [31]	N=92/cross-sectional	Mixed	NA	ASDA/Median 60.5 (40.25–73.0.25.0)	Health anxiety↑	↑: Health anxiety (P=0.007)
Webb et al. (2024)/Australia[32]	N=436/cross-sectional	Mixed	NA	DABBS/NA	Fear of cancer recurring or progressing↑	↑: Fear of cancer recurrence or progression (r=0.440, P<0.01).

*DA *Death anxiety, *TDAS *Templer’s Death Anxiety Scale, *QoL *Quality of life, *DAQ *Death Anxiety Questionnaire, *TPDAS *Thorson-Powell’s Death Anxiety Scale, *CL-FODS *Collett–Lester Fear of Death Scale, *FC *Family caregivers; Level of activities of daily living, *ADL DADDS-CG *Death and Dying Distress Scale for Caregivers, *ASDA *Arabic Scale of Death Anxiety, *CIO *Cancer information overload, *DABBS *Death Anxiety Beliefs and Behaviours Scale, ↑:Positive correlation; ↓: Negative correlation ↗: Mediating effect; CI: Confidence Interval

### Studies methodological quality assessment

All studies used an adequate sampling frame to address the target population and provided a detailed description of their sample and context. All included studies employed appropriate research methods to answer the research question, used validated scales to measure the different variables, and applied appropriate statistical analyses. The results of the assessment are presented in Supplementary File 2.

### Study characteristics

In total, 4,792 family caregivers were included across the 18 studies. Per-study sample sizes ranged from 36 to 588 participants. Gender was reported in all studies: 2,148 men (44.8%), 2,639 women (55.1%), and 5 identifying as other (0.1%). All studies adopted a cross-sectional design. Regarding cancer types among the included studies, 13 studies involved caregivers of patients with mixed cancer types, two focused on brain tumor, one on lung cancer, one on gynecologic cancer, and one on gastric cancer. With respect to cancer staging, seven studies included patients across stages I to IV, four studies focused on stages III to IV, one study included only one stage IV cancer, one study involved patients with advanced cancer, and one with terminal cancer. Four studies did not specify staging. Regarding geographic distribution, the studies were conducted in China (*n* = 8; 2687 participants), Turkey (*n* = 3; 718 participants), the United States (*n* = 3; 155 participants), Iran (*n* = 2; 656 participants), Australia (*n* = 1; 436 participants), and Germany (*n* = 1; 140 participants). Detailed study characteristics are provided in Table 1.

### Death anxiety levels and measurement

Eleven studies used Templer’s Death Anxiety Scale (T-DAS) [8, 11, 12, 14, 15, 17, 25–29]. Two studies employed the Death and Dying Distress Scale for Caregivers (DADDS-CG) [18, 30]. One study used Thorson-Powell’s Death Anxiety Scale [20], one used the Death Anxiety Questionnaire (DAQ) [21], one used the Arabic Scale of Death Anxiety (ASDA) [31], one used the Death Anxiety Beliefs and Behaviors Scale (DABBS) [32], and one used the Collett–Lester Fear of Death Scale (CL-FODS) [19].

Death anxiety scores for family caregivers were reported in 16 of the 18 included studies. Among the 11 studies using T-DAS, seven conducted in China [8, 11, 12, 17, 25, 26, 28] and two in Iran [15, 27] consistently found caregivers scored above the scale midpoint, indicating elevated levels of death anxiety; Iranian caregivers generally scored higher than their Chinese counterparts. By contrast, a U.S. study using T-DAS reported scores below the scale midpoint [29], a finding echoed by another U.S. study using DAQ [21].

Two studies employing the DADDS-CG reported mean (SD) scores indicating moderate distress: Germany 39.2 (18.5) [18] and the United States 34.81 (18.96) [30], with the German sample scoring slightly higher. A Turkish study using the Thorson-Powell Death Anxiety Scale reported that caregivers of gynecologic cancer patients had mild levels of death anxiety [20], while another Turkish study using the ASDA found median scores slightly above the scale midpoint [31].

Death anxiety among family caregivers varied by cultural context: caregivers in China generally reported moderate to high levels of death anxiety, with Iranian caregivers showing slightly higher levels. Caregivers in Turkey showed mild-to-moderate levels, while those in the United States consistently exhibited low-to-moderate levels. In Germany, caregivers exhibited moderate levels.

### Factors related to death anxiety

Several factors were found to be significantly associated with death anxiety among family caregivers (*p* < 0.05). These were categorized into the following six clusters: (a) personal factors, (b) disease- and caregiving-related factors, (c) psychosocial factors, (d) self-regulatory factors, (e) other factors, (f) actor-partner effects. Detailed results are provided in Table 2.

**Table 2:** Results

Number	Factors/Variables	Studies (Representative)	Description (Effect on DA Outcome)
	Personal factors		
1	Female	[8, 11, 12, 14, 15, 17, 18, 27, 30]	↑
2	Age	[11, 12, 14, 15, 19]	-
3	Having religious beliefs or higher prayer frequency	[8, 15, 17]	↓
4	Death Education	[19, 25]	-
5	Educational Level (Higher)	[8, 11, 15, 18, 19]	-
6	Caregiver's Poor Health Status	[8, 12]	↑
7	Place of residence (urban areas)	[19]	↓
8	Family structure	[11, 15, 19]	-
	Married	[19]	↑
	Having siblings	[14]	↑
9	Economic Factors (Lower Income/Financial Burden/Lower medical reimbursement rates)	[8, 11, 14, 15, 19]	↑
10	Having bereavement experience	[12]	↑
11	Personality Traits (Neuroticism/Trait Anxiety)	[8, 17, 26]	↑
	Disease- and caregiving-related Factors		
12	Disease Recurrence/Fear of Recurrence	[8, 11, 12, 30]	↑
13	Patient's Lower Functional Status	[10, 12, 17]	↑
14	Advanced Cancer Stage (III-IV)	[19, 30]	↑
Number	Factors/Variables	Studies	Effect on DA Outcome/Relationship with DA
15	Longer Caregiving/Illness Duration	[17, 25]	↑
16	Unstable disease condition	[8]	↑
17	Lack of external support/absence of palliative care/higher subjective caregiving burden	[8, 10, 25]	↑
18	Receiving surgery or chemotherapy	[15, 17]	↓
19	Patients were fully aware of their cancer diagnosis	[11]	↑
20	Liver cancer	[19]	↑
	Psychosocial Factors		
21	Higher quality of life	[15, 17, 21, 25, 26, 28]	↓
22	Higher social support	[8, 17, 20, 26]	↓
	Self-regulatory Factors		
23	Higher self-efficacy	[8, 11]	↓
24	Positive coping style (Positive vs. Maladaptive)	[11, 19]	↓
25	Higher self-regulatory fatigue	[12]	↑
	Other factors		
26	Posttraumatic growth/Death anxiety in patients/Health anxiety	[14, 25, 31]	↑
	Actor-partner effects		
27	Dependency/Self-control/Achievement	[28]	↑: Caregiver achievement and dependency,Patient achievement and dependency.↓:Caregiver/Patient self‑control
28	Patients’ fear of cancer recurrence	[29]	Increase

↑:Increase; ↓: Decrease; -: Inconsistency

### Personal factors

#### Gender

A total of nine studies reported the association between caregiver gender and death anxiety. These found that female caregivers consistently experienced significantly higher levels of death anxiety than males [8, 11, 12, 14, 15, 17, 18, 27, 30].

#### Age

Five studies explored the association between caregiver age and death anxiety, with mixed findings [11, 12, 14, 15, 19]. Xie et al. found that older family caregivers (60–78 years) had the highest levels of death anxiety [19]. In contrast, two studies found that younger caregivers (18–44 years) exhibited greater death anxiety [12, 15]. Another study indicated that middle-aged caregivers (36–59 years) experienced higher levels of death anxiety than other age groups [11]. Care recipient age was also found to be relevant: caregivers of older patients reported higher levels of death anxiety [14].

#### Religion-related factors

Three studies indicated that religious belief and practices acted as protective factors [8, 15, 17]. Caregivers with religious beliefs had significantly lower death anxiety scores than those without [8, 17]. Furthermore, Soleimani et al. [15] reported that higher frequency of religious practice (e.g. daily prayer) was associated with reduced death anxiety.

#### Death education

Two studies investigated the impact of death education on caregiver death anxiety, yielding inconsistent findings [19, 25]. One study found that family caregivers who had received death education had lower death anxiety [19]. However, another study reported that caregivers who had received death education exhibited higher death anxiety [25].

#### Educational level

Regarding the association between educational level and death anxiety, the synthesized evidence revealed inconsistent findings [8, 11, 15, 18, 19]. While four studies consistently indicated that higher educational attainment among family caregivers was associated with lower levels of death anxiety [8, 11, 18, 19], one study by Soleimani et al. [15] reported a contradictory association, noting that caregivers with a high school education or above exhibited higher levels of death anxiety.

#### Health status of caregivers

Three studies highlighted the role of caregivers’ physical and mental health. Caregivers with chronic illnesses and poorer self-rated health reported higher death anxiety [8, 12]. For example, caregivers with a history of physical trauma or chronic diseases demonstrated significantly elevated death anxiety scores [8, 12].

#### Place of residence

One study found that caregivers residing in counties or towns had higher death anxiety scores compared with those in urban areas [19].

#### Family structure

Three studies investigated the role of family structure in caregiver death anxiety. Liu [11] found that parents of patients reported the highest levels of death anxiety, while Soleimani et al. observed that daughters had higher death anxiety compared with other caregiver roles [15]. Xie et al. [19] reported that spouses exhibited the highest death anxiety, and that married caregivers had significantly higher death anxiety than unmarried or divorced caregivers. In addition, one study found that caregivers with siblings had higher levels of death anxiety than those without [14]. Other studies reported no significant differences by family role.

#### Economic factors

Five studies demonstrated a negative association between income level and death anxiety [8, 11, 14, 15, 19], indicating that caregivers with lower income reported higher death anxiety. A further study indicated that a lower medical insurance reimbursement rate for patients was significantly associated with higher levels of death anxiety among caregivers, suggesting that the financial burden of out-of-pocket medical expenses may exacerbate psychological distress [8]. Additionally, research led by Xie et al. [19] found that caregivers who reported greater difficulty in affording treatment exhibited higher death anxiety scores.

Employment status was also significant. Two studies found that employed family caregivers had lower death anxiety scores than those unemployed. However, Liu et al. reported that full-time caregivers reported higher levels of death anxiety compared with those providing part-time care [11].

#### Bereavement experience

One study reported that family caregivers with bereavement experience had higher levels of death anxiety than those without such experience [12].

#### Personality traits

Three studies indicated that death anxiety was associated with personality traits. Trait anxiety was positively associated with death anxiety, such that caregivers with higher trait anxiety scores experienced greater death anxiety [17, 26]. Neuroticism was also positively correlated with death anxiety, whereas extraversion was negatively correlated, suggesting a protective effect [8].

### Disease- and caregiving-related factors

Four studies indicated that death anxiety was associated with factors related to disease recurrence (e.g., experience of recurrence, site of recurrence, fear of recurrence) [8, 11, 12, 30]. Caregivers of patients with recurrent disease consistently reported higher death anxiety, with greater levels observed when recurrence involved distant metastasis compared with local recurrence [12]. Willis et al. further demonstrated a positive correlation between family caregivers’ fear of recurrence and death anxiety [30].

Care recipient functional status was another key factor: lower patient self-care ability was associated with higher death anxiety [10, 12, 17]. Similarly, advanced tumor stage (stages III-IV) was significantly linked to higher death anxiety [19, 30]. Additionally, other disease- and caregiving-related variables included: longer care duration [17], longer illness duration [25], shorter expected survival period [8], unstable disease condition [8], history of resuscitation experiences [8], absence of palliative care [8], lack of external support [25], and higher subjective caregiving burden [10].

Treatment modality also played a role. Family caregivers of patients actively receiving surgery or chemotherapy reported lower death anxiety than those whose patients were not undergoing treatment or receiving radiotherapy [15, 17]. Interestingly, one study reported that caregivers experienced the highest death anxiety when patients were fully aware of their cancer diagnosis [11].

Only one study suggested that the type of cancer influenced death anxiety levels among family caregivers. Xie et al. reported that caregivers of liver cancer patients had the highest death anxiety[19]. However, most other studies found no significant differences in anxiety levels across different cancer types.

### Psychosocial factors

Six studies indicated that quality of life was a significant correlate of death anxiety. Lower quality of life scores were consistently associated with higher levels of caregiver death anxiety [15, 17, 21, 25, 26, 28]. One study revealed both direct and indirect effects of quality of life, mediated through social support and personality traits [26]. Additionally, Lau et al. found that caregivers’ death anxiety was negatively associated with both their own quality of life and that of the patients [28].

Four studies confirmed that social support was negatively associated with death anxiety, meaning that caregivers with strong support networks reported lower levels of death anxiety [8, 17, 20, 26].

### Self-regulatory factors

Two studies indicated a negative association between self-efficacy and death anxiety, suggesting that higher self-efficacy was protective [8, 11].

Coping strategies were also significant. Caregivers who adopted positive coping styles reported lower death anxiety, whereas reliance on maladaptive coping was associated with higher anxiety [11, 19].

Additionally, one study revealed that self-regulatory fatigue was positively correlated with death anxiety, while positive psychological aspects were negatively correlated, indicating a dual role of regulatory processes in shaping caregiver outcomes [12].

### Other factors

Death anxiety appeared to exert reciprocal effects on related psychological variables. Specifically, one study indicated that higher levels of death anxiety in patients were associated with higher death anxiety scores among their caregivers [25]. Posttraumatic growth was positively correlated with death anxiety [14]. Moreover, Eraslan & İlhan reported that death anxiety significantly predicted higher levels of health anxiety among caregivers [31].

### Actor-partner effects

Two studies utilized the Actor-Partner Interdependence Model (APIM) to examine dyadic effects [28, 29]. Lau et al. [28] demonstrated that dysfunctional attitudes in both caregivers and patients influenced caregiver death anxiety. Specifically, one study demonstrated that different dimensions of dysfunctional attitudes were correlated with death anxiety in distinct ways [28]. For example, higher levels of dependency in both patients and caregivers were associated with increased death anxiety in caregivers. Conversely, greater self-control in either patients or caregivers was linked to lower levels of death anxiety in caregivers. Regarding the achievement dimension of dysfunctional attitudes, death anxiety in family caregivers was positively correlated with their own achievement orientation but negatively correlated with that of their patients [28].

Braun et al. further showed that patients’ fear of cancer recurrence was positively associated with caregiver death anxiety, highlighting the interdependent nature of patient-caregiver psychological processes [29].

## Discussion

This systematic review identified multiple factors associated with death anxiety among family caregivers of cancer patients, encompassing personal characteristics, disease and caregiving-related variables, psychosocial resources, and self-regulatory capacities. Consistent patterns were observed for gender, education, economic status, and personality traits, whereas findings regarding age, religious involvement, death education, family structure, place of residence, and bereavement experience were mixed.

### Impact of personal factors

Female caregivers consistently reported higher death anxiety than males, a finding consistent with prior literature on gendered differences in death attitudes [11, 14, 33]. Explanations may include women’s greater emotional empathy, compounded caregiving responsibilities, and lower stress resilience. Similarly, caregivers with unstable economic conditions were more vulnerable, likely due to limited health literacy, financial strain, and reduced access to resources [8, 11, 14, 15, 34–36]. Personality traits also played a role: neuroticism and trait anxiety increased susceptibility, whereas extraversion appeared protective [8, 26, 37, 38]. These findings highlight the need for psychosocial screening and differentiated psychological support for caregivers at risk.

By contrast, the influence of age, religion, educational attainment level and death education remains inconclusive. Some studies linked younger or middle-aged caregivers to higher death anxiety, while others pointed to older age [11, 12, 14, 15, 19]. Religious belief and prayer frequency were sometimes protective [8, 15, 17], but not consistently so. The variation in findings may be attributed to the distinct views on life and death held by different religions, combined with the absence of a standardized definition of religious belief and a lack of precise screening for devoutness, factors which could have influenced the observed results [39, 40]. Similarly, educational attainment level and death education showed contradictory effects, possibly reflecting taboos around death discussions in educational and cultural settings [19]. More rigorous, longitudinal research is required to clarify these associations.

### Disease- and caregiving-related factors

Caregiving context and patient characteristics strongly shaped caregiver death anxiety. Higher tumor stage, disease recurrence, lower patient self-care ability, and longer caregiving duration consistently predicted higher anxiety [8, 11, 12, 17, 19, 25, 30]. Lack of palliative care and insufficient social support further intensified caregiver distress [8, 25]. Importantly, caregivers reported greater anxiety when patients were fully aware of their diagnosis, likely due to the dual emotional burden of supporting both patient and self [11].

These findings underscore the importance of integrating family-centered psychosocial interventions into oncology and palliative care, including routine assessment of caregiver burden, psychoeducation around disease recurrence, and structured support for sharing care responsibilities within families [8, 25].

### Psychosocial and self-regulatory factors as protective mechanisms

Consistent with stress-buffering theory [41], higher quality of life and greater social support emerged as robust protective factors [8, 15, 17, 21, 25, 28]. Conversely, impaired quality of life and lack of support amplified anxiety, suggesting a vicious cycle of distress, reduced coping capacity, and deteriorating well-being. Self-efficacy and positive coping styles also mitigated death anxiety [8, 11, 19], whereas self-regulatory fatigue heightened vulnerability [12]. These findings support interventions grounded in positive psychology and self-efficacy enhancement [42, 43], aimed at equipping caregivers with adaptive coping strategies and emotional resilience.

### The dual role of death anxiety

Notably, death anxiety may exert both negative and adaptive effects. On the one hand, it predicted heightened health anxiety and psychological distress [44]. On the other hand, it was linked to post-traumatic growth, suggesting that confronting mortality can catalyze meaning-making and resilience [14, 44]. This dual role highlights the complexity of death anxiety as both a burden and a potential driver of personal growth. Future longitudinal studies should explore these pathways across different cultural and clinical contexts.

#### Necessity of a dyadic perspective in caregiving

Most research on caregiver death anxiety examines patients or caregivers in isolation, typically emphasizing demographics or individual correlates. This one-sided approach risks bias and overlooks the interdependence within patient-caregiver dyads. Prior work already shows that death anxiety levels may differ between patients and caregivers—and the direction of that difference varies by context. For example, caregivers of brain tumor patients reported slightly higher death anxiety than patients, and caregiver death anxiety predicted patients’ fear of recurrence [29], whereas other studies observed comparable death anxiety levels across the dyad [28].

Evidence also suggests that psychological factors are bidirectionally linked. Patients’ dysfunctional attitudes, quality of life, fear of recurrence, and death anxiety directly influenced caregiver outcomes, and vice versa [28, 29]. Domain-specific effects were observed: for example, dependency in caregivers and achievement orientation in patients were positively correlated with caregiver death anxiety, whereas self-control in either member of the dyad was protective. These findings are consistent with other studies of caregiver vulnerability [19].

Taken together, these data support treating the patient and primary caregiver as an integrated unit of care. Future studies should routinely adopt dyadic designs, such as the APIM, to disentangle actor effects (one’s own characteristics on one’s death anxiety) from partner effects (the other’s characteristics on one’s death anxiety). Comprehensive exploration of both psychological and physical perspectives within families will provide a stronger empirical foundation for family-centered assessment and intervention in cancer care.

### Limitations

This review has several limitations. First, the number of eligible studies was modest, and most samples involved mixed cancer types, limiting conclusions about diagnosis-specific correlates. Second, the predominance of cross-sectional designs precludes causal inference regarding antecedents and consequences of caregiver death anxiety. Third, heterogeneity in measurement instruments and potential cultural variability (e.g., scoring distributions, cut points) may have introduced unmeasured heterogeneity. Fourth, restricting inclusion to English and Chinese publications may have introduced language bias. Finally, unreported or inconsistent adjustment for confounders (e.g., socioeconomic status, disease severity, treatment status) could affect observed associations. Future work should use longitudinal, adequately powered, culturally sensitive designs; test measurement invariance across instruments/contexts; and employ analytic strategies capable of modeling trajectories and causal pathways.

## Conclusion

This systematic review synthesizes evidence on caregiver death anxiety after a cancer diagnosis and its correlates across six domains: personal, disease-/care-related, psychosocial, self-regulatory, other, and actor-partner effects. Psychosocial resources, higher social support and better quality of life, consistently functioned as protective factors. Self-regulatory capacities, greater self-efficacy, positive coping, and positive psychological aspects, were also protective, whereas self-regulatory fatigue, fear of progression/recurrence, and greater caregiving burden/stress were associated with elevated death anxiety. Under some conditions, death anxiety co-occurred with post-traumatic growth, suggesting a potential adaptive dimension.

Findings for several demographic and disease-related variables (e.g., age, religious involvement, death education, family structure) were inconsistent, underscoring the need for rigorous research to clarify when and for whom these factors matter. We recommend longitudinal, dyadic studies with sufficient sample sizes, culturally sensitive measurement, and models such as APIM to identify mutual influence mechanisms and temporal dynamics, thereby informing family-centered interventions.

In nursing practice, family caregivers often experience death anxiety during long-term cancer care. Therefore, reducing caregivers’ death anxiety should be incorporated as a routine nursing goal. Clinical teams are advised to integrate the following into oncology care protocols: routine screening of death anxiety and its risk factors among caregivers; family-centered communication and psychoeducation; early identification of high-risk individuals and provision of tailored interventions such as social support, coping skills training, and self-efficacy enhancement. Through these strategies, caregivers’ mental health can be safeguarded, enabling them to better adapt to the cancer crisis and persistent caregiving tasks.

### Declaration of generative AI and AI-assisted technologies in the writing process

ChatGPT was used for partial translation of the manuscript to improve language clarity and readability. All translated content was subsequently reviewed, revised, and approved by the corresponding author. The authors take full responsibility for the accuracy, integrity, and originality of the final manuscript.

## Supplementary Information

Supplementary Material 1

Supplementary Material 2

## Data Availability

The data used in this systematic review were obtained from published studies available through PubMed, Embase, PsycINFO, Scopus, Web of Science, MEDLINE, CNKI, as outlined in the methods section. All references are included in the manuscript. No additional datasets were generated or analyzed during this study.
