# Supplementary material for: Factors associated with death anxiety in family caregivers of cancer patients: a systematic review
Source: BMC Palliat Care. 2026 Apr 30;25:175. doi: 10.1186/s12904-026-02127-8 (PMC13273968; doi:10.1186/s12904-026-02127-8)
Supplement: Supplementary file 1 — Supplementary Material 1 [file 12904_2026_2127_MOESM1_ESM.docx]

| **Database** | **Search strategy** |
| --- | --- |
| Pubmed | ("death depression"[Title/Abstract] OR "death anxiety"[Title/Abstract]) AND ("Caregivers"[Title/Abstract] OR "care givers"[Title/Abstract] OR "Carers"[Title/Abstract] OR "family caregivers"[Title/Abstract] OR "spouse caregivers"[Title/Abstract] OR "informal caregivers"[Title/Abstract]) AND ("Neoplasia"[Title/Abstract] OR "Neoplasias"[Title/Abstract] OR "Neoplasm"[Title/Abstract] OR "Tumors"[Title/Abstract] OR "Tumor"[Title/Abstract] OR "Cancer"[Title/Abstract] OR "Cancers"[Title/Abstract] OR "Malignancy"[Title/Abstract] OR "Malignancies"[Title/Abstract] OR "malignant neoplasms"[Title/Abstract] OR "malignant neoplasm"[Title/Abstract] OR "neoplasm malignant"[Title/Abstract] OR "neoplasms malignant"[Title/Abstract] OR "benign neoplasms"[Title/Abstract] OR "neoplasms benign"[Title/Abstract] OR "benign neoplasm"[Title/Abstract] OR "neoplasm benign"[Title/Abstract]) |
| Web of Science | #1：TS=(death depression OR death anxiety)  #2：TS=(Caregivers OR care givers OR Carers OR family caregivers OR spouse caregivers OR informal caregivers)  #3: TS=(Neoplasia OR Neoplasias OR Neoplasm OR Tumors OR Tumor OR Cancer OR Cancers OR Malignancy OR Malignancies OR malignant neoplasms OR neoplasm malignant OR neoplasms malignant OR benign neoplasms OR neoplasms benign OR benign neoplasm OR neoplasm benign OR malignant neoplasm)  #4：#1 AND #2 AND #3 |
| MEDLINE | #1：TS=(death depression OR death anxiety)  #2：TS=(Caregivers OR care givers OR Carers OR family caregivers OR spouse caregivers OR informal caregivers)  #3: TS=(Neoplasia OR Neoplasias OR Neoplasm OR Tumors OR Tumor OR Cancer OR Cancers OR Malignancy OR Malignancies OR malignant neoplasms OR neoplasm malignant OR neoplasms malignant OR benign neoplasms OR neoplasms benign OR benign neoplasm OR neoplasm benign OR malignant neoplasm)  #4：#1 AND #2 AND #3 |
| Scopus | ( TITLE-ABS-KEY ( death depression ) OR TITLE-ABS-KEY ( death anxiety ) AND TITLE-ABS-KEY ( Caregivers ) OR TITLE-ABS-KEY ( care givers ) OR TITLE-ABS-KEY ( Carers ) OR TITLE-ABS-KEY ( family caregivers ) OR TITLE-ABS-KEY ( spouse caregivers ) OR TITLE-ABS-KEY ( informal caregivers ) AND TITLE-ABS-KEY ( Neoplasia ) OR TITLE-ABS-KEY ( Neoplasias ) OR TITLE-ABS-KEY ( Neoplasm ) OR TITLE-ABS-KEY ( Tumors ) OR TITLE-ABS-KEY ( Tumor ) OR TITLE-ABS-KEY ( Cancer ) OR TITLE-ABS-KEY ( Cancers ) OR TITLE-ABS-KEY ( Malignancy ) OR TITLE-ABS-KEY ( Malignancies ) OR TITLE-ABS-KEY ( malignant neoplasms ) OR TITLE-ABS-KEY ( neoplasm malignant ) OR TITLE-ABS-KEY ( neoplasms malignant ) OR TITLE-ABS-KEY ( benign neoplasms ) OR TITLE-ABS-KEY ( neoplasms benign ) OR TITLE-ABS-KEY ( benign neoplasm ) OR TITLE-ABS-KEY ( neoplasm benign ) OR TITLE-ABS-KEY ( malignant neoplasm ) ) |
| Embase | #1：'death depression':ti,ab,kw OR 'death anxiety':ti,ab,kw  #2：caregivers:ti,ab,kw OR 'care givers':ti,ab,kw OR carers:ti,ab,kw OR 'family caregivers':ti,ab,kw OR 'spouse caregivers':ti,ab,kw OR 'informal caregivers':ti,ab,kw  #3：neoplasia:ti,ab,kw OR neoplasias:ti,ab,kw OR neoplasm:ti,ab,kw OR tumors:ti,ab,kw OR tumor:ti,ab,kw OR cancer:ti,ab,kw OR cancers:ti,ab,kw OR malignancy:ti,ab,kw OR malignancies:ti,ab,kw OR 'malignant neoplasms':ti,ab,kw OR 'neoplasm malignant':ti,ab,kw OR 'neoplasms malignant':ti,ab,kw OR 'benign neoplasms':ti,ab,kw OR 'neoplasms benign':ti,ab,kw OR 'benign neoplasm':ti,ab,kw OR 'neoplasm benign':ti,ab,kw OR 'malignant neoplasm':ti,ab,kw  #4： #1 AND #2 AND #3 |
| PsycInfo | **Abstract**: death depression *OR* **Abstract**: death anxiety *AND* **Abstract**: caregivers *OR* **Abstract**: caregivers *OR* **Abstract**: Carers *OR* **Abstract**: family caregivers *OR* **Abstract**: spouse caregivers *OR* **Abstract**: informal caregivers *AND* **Abstract**: Neoplasia *OR* **Abstract**: Neoplasias *OR* **Abstract**: Neoplasm *OR* **Abstract**: Tumors *OR* **Abstract**: tumor *OR* **Abstract**: cancers *OR* **Abstract**: cancer *OR* **Abstract**: Malignancy *OR* **Abstract**: Malignancies *OR* **Abstract**: malignant neoplasms *OR* **Abstract**: malignant neoplasm *OR* **Abstract**: Neoplasm Malignant *OR* **Abstract**: Neoplasms Malignant *OR* **Abstract**: benign neoplasms *OR* **Abstract**: Neoplasms Benign *OR* **Abstract**: benign neoplasm *OR* **Abstract**: Neoplasm Benign |
| CNKI | (篇关摘:癌症 + 癌+ 肿瘤 +瘤(精确))AND(篇关摘:家庭照顾者 + 家属 + 照顾者 +配偶(精确))AND(篇关摘:死亡焦虑 + 死亡抑郁(精确)) |
