# Supplementary material for: Factors associated with death anxiety in family caregivers of cancer patients: a systematic review
Source: BMC Palliat Care. 2026 Apr 30;25:175. doi: 10.1186/s12904-026-02127-8 (PMC13273968; doi:10.1186/s12904-026-02127-8)
Supplement: Supplementary file 2 — Supplementary Material 2 [file 12904_2026_2127_MOESM2_ESM.docx]

**Analytical cross sectional studies Critical Appraisal Tool**

| **Author/ Year of publication** | **1. Were the criteria for inclusion in the sample clearly defined?** | **Were the study subjects and the setting described in detail?** | **3. Was the exposure measured in a valid and reliable way?** | **4. Were objective, standard criteria used for measurement of the condition?** | **5. Were confounding factors identified?** | **6. Were strategies to deal with confounding factors stated?** | **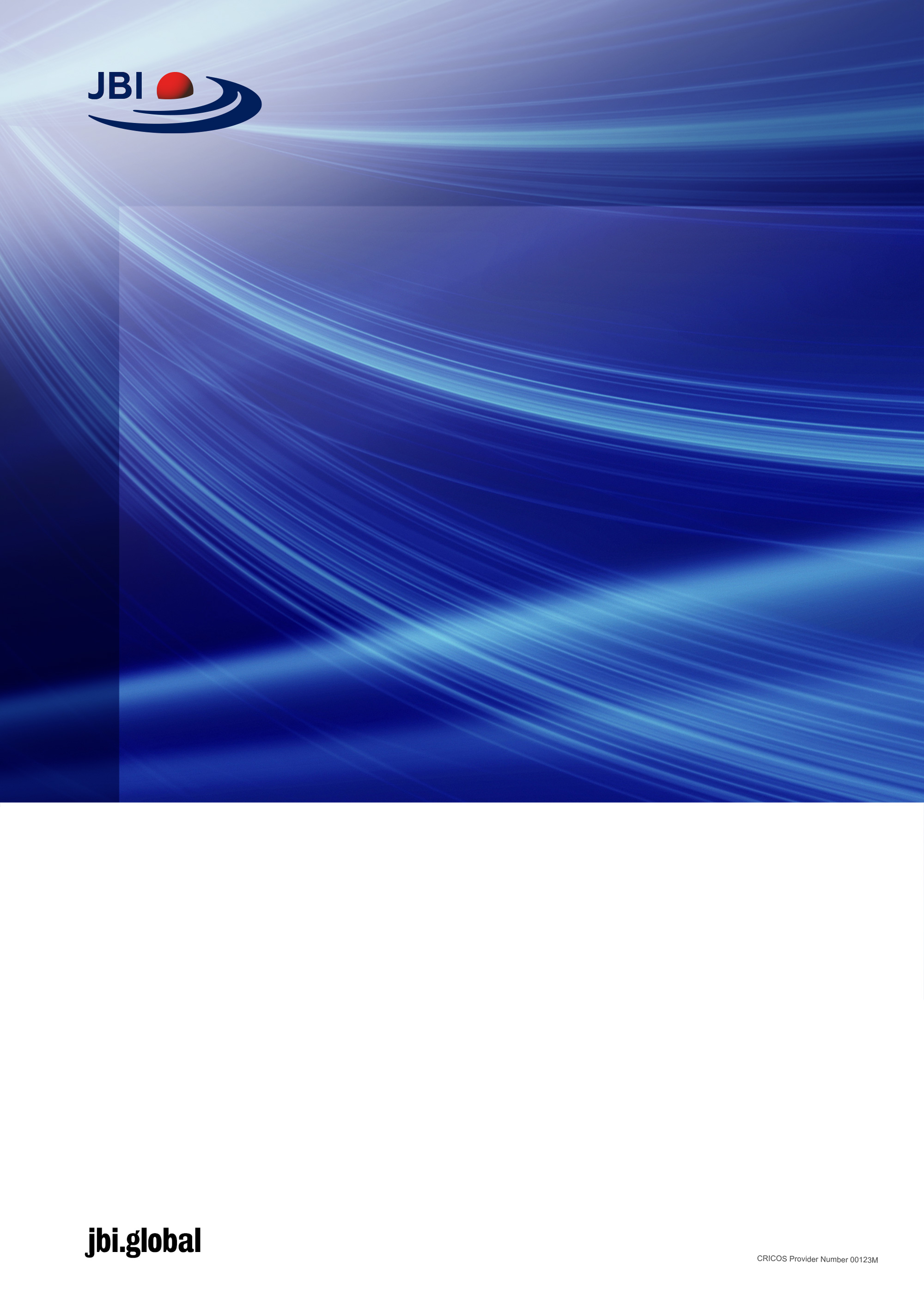7. Were the outcomes measured in a valid and reliable way?** | **8. Was appropriate statistical analysis used?** | **Score** | **Risk of****bias** |
| --- | --- | --- | --- | --- | --- | --- | --- | --- | --- | --- |
| Qian (2022) | yes | yes | yes | yes | yes | yes | yes | yes | 100% | Low |
| Li et al. (2024) | yes | yes | yes | yes | yes | yes | yes | yes | 100% | Low |
| Liu (2023) | yes | yes | yes | yes | yes | yes | yes | yes | 100% | Low |
| Liu et al. (2021) | yes | yes | yes | yes | yes | yes | yes | yes | 100% | Low |
| Soleimani et al. (2017) | yes | yes | yes | yes | yes | yes | yes | yes | 100% | Low |
| Bu, et al. (2024) | yes | yes | yes | yes | yes | yes | no | yes | 100% | Low |
| Ying et al. (2024) | yes | yes | yes | yes | yes | yes | yes | yes | 100% | Low |
| Walbaum et al. (2024) | yes | yes | yes | yes | yes | yes | yes | yes | 100% | Low |

| **Author/ year of publication** | **1. Were the criteria for inclusion in the sample clearly defined?** | **2. Were the study subjects and the setting described in detail?** | **3. Was the exposure measured in a valid and reliable way?** | **4. Were objective, standard criteria used for measurement of the condition?** | **5. Were confounding factors identified?** | **6. Were strategies to deal with confounding factors stated?** | **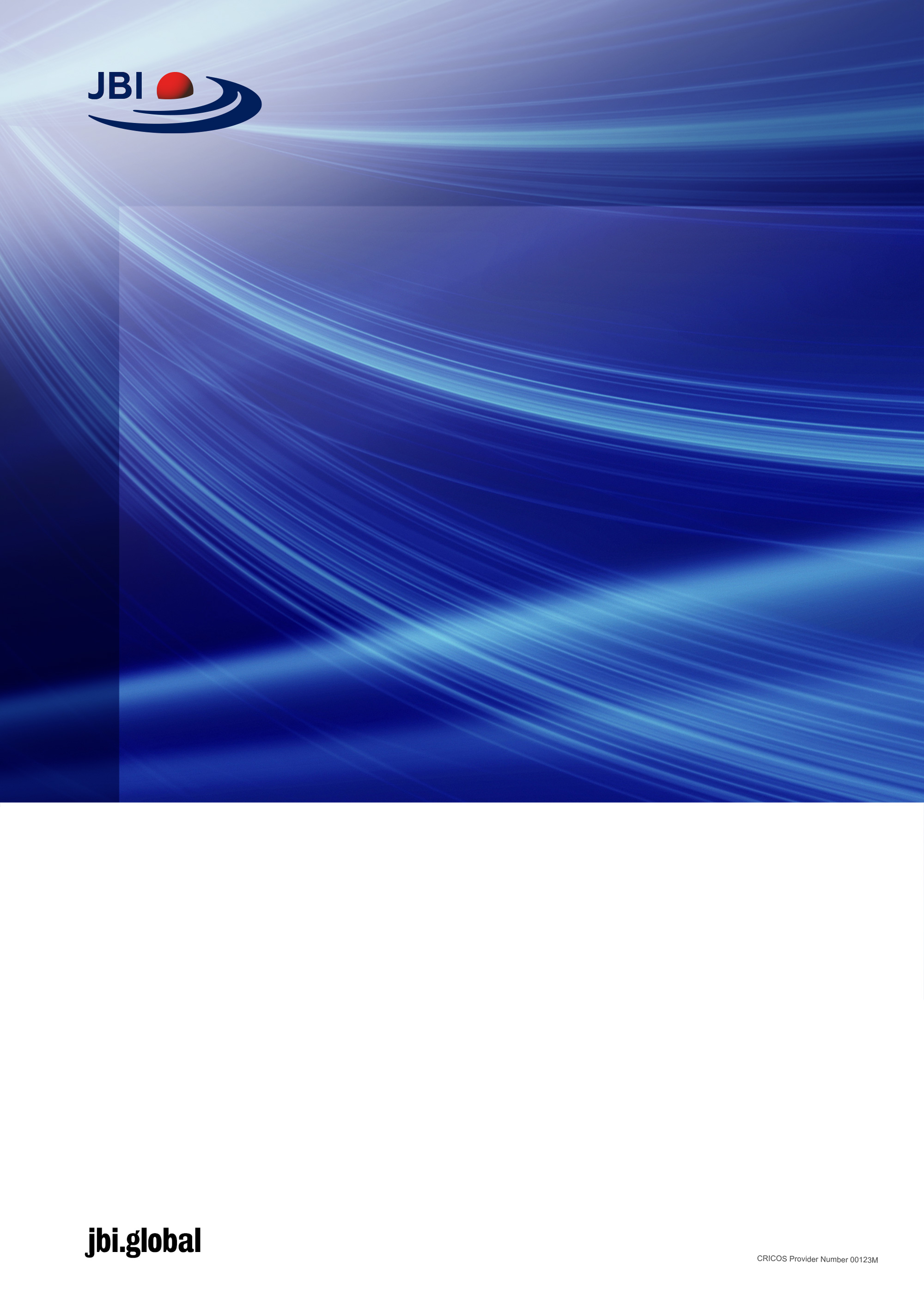7. Were the outcomes measured in a valid and reliable way?** | **8. Was appropriate statistical analysis used?** | **Score** | **Risk of****bias** |
| --- | --- | --- | --- | --- | --- | --- | --- | --- | --- | --- |
| Xie et al. (2025) | yes | yes | yes | yes | yes | yes | yes | yes | 100% | Low |
| Sherman et al. (2010) | yes | yes | yes | yes | yes | no | yes | yes | 87.5% | Low |
| Uslu-Sahan et al. (2019) | yes | yes | yes | yes | no | no | yes | no | 75% | Low |
| Alkan et al. (2020) | yes | yes | yes | yes | yes | yes | yes | yes | 100% | Low |
| Soleimani et al. (2016) | yes | yes | yes | yes | yes | no | yes | yes | 87.5% | Low |
| Lau et al. (2018) | yes | yes | yes | yes | yes | yes | yes | yes | 100% | Low |
| Braun et al. (2021) | yes | yes | yes | yes | yes | no | yes | yes | 87.5% | Low |
| Willis et al. (2023) | yes | yes | yes | yes | yes | no | yes | yes | 87.5% | Low |
| **Author/ year of publication** | **1. Were the criteria for inclusion in the sample clearly defined?** | **2. Were the study subjects and the setting described in detail?** | **3. Was the exposure measured in a valid and reliable way?** | **4. Were objective, standard criteria used for measurement of the condition?** | **5. Were confounding factors identified?** | **6. Were strategies to deal with confounding factors stated?** | **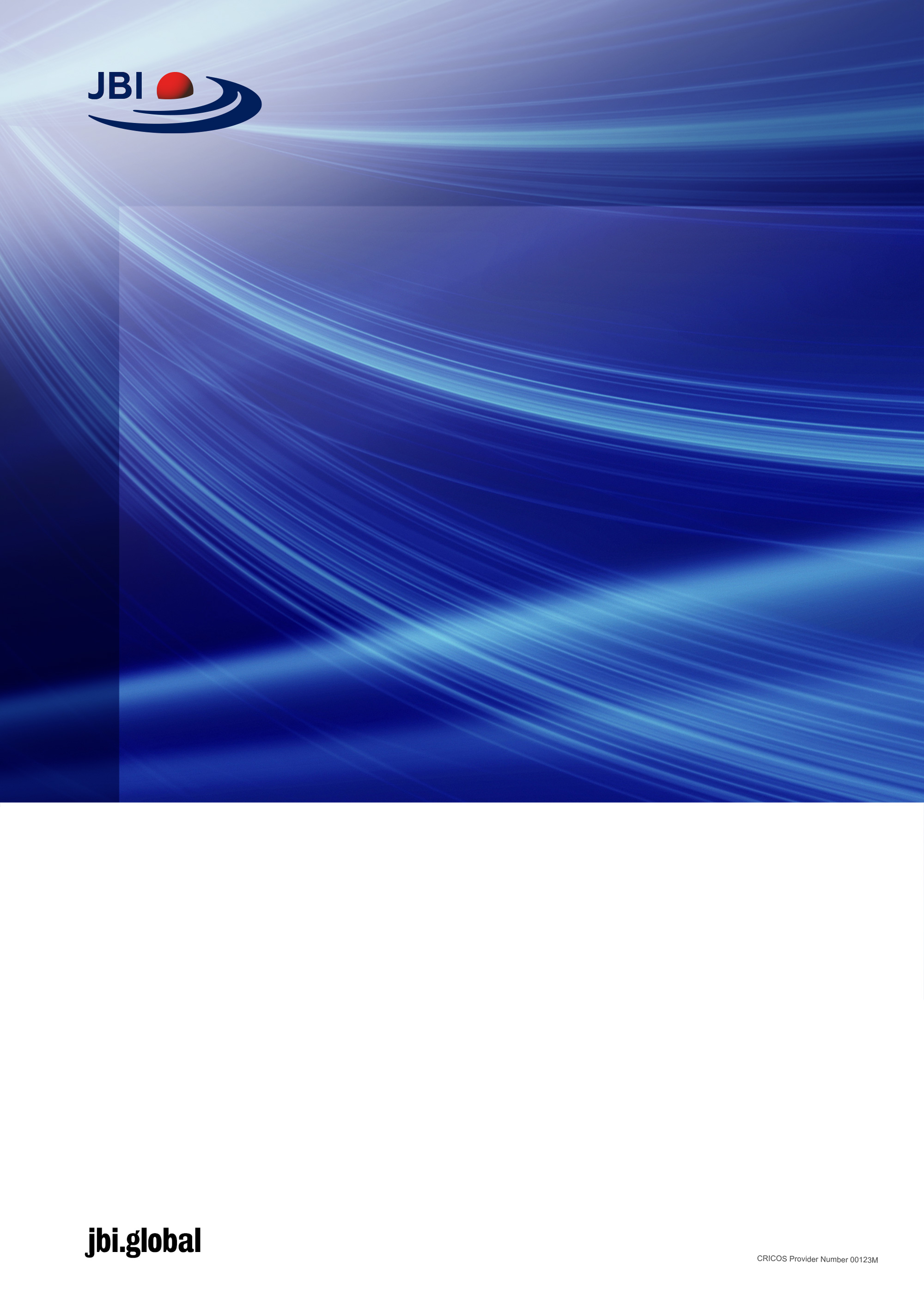7. Were the outcomes measured in a valid and reliable way?** | **8. Was appropriate statistical analysis used?** | **Score** | **Risk of****bias** |
| Eraslan, P & İlhan, A. (2023) | yes | yes | yes | yes | yes | yes | yes | yes | 100% | Low |
| Webb et al. (2024) | yes | yes | yes | yes | yes | no | yes | yes | 87.5% | Low |
